# When Empiric Therapy Fails: Clinical and Economic Consequences in uUTIs Among US Women

**DOI:** 10.3390/microorganisms14091993

**Published:** 2026-09-09

**Authors:** Debra L. Fromer, Jeffrey J. Ellis, Rose Chang, Meghan E. Luck, Megan Pinaire, Malena Mahendran, Madison Preib, Mei Sheng Duh, Amy Edgecomb

**Affiliations:** 1 Hackensack University Medical Center, Hackensack, NJ 07601, USA; 2 GSK, Collegeville, PA 19426, USA; 3 Analysis Group, Inc., Boston, MA 02199, USA

**Keywords:** clinical outcomes, economic burden, healthcare resource utilization, antibiotic treatment failure, uncomplicated urinary tract infection

## Abstract

Uncomplicated urinary tract infection (uUTI) management is increasingly challenging due to antibiotic treatment failure (TF). This retrospective, observational cohort study used Optum’s de-identified Electronic Health Record dataset (January 2017–September 2022) to investigate the impact of antibiotic TF (defined as a second oral antibiotic prescription, administration of an intravenous antibiotic, or an emergency department visit/hospitalization with a new primary diagnosis of urinary tract infection <28 days post index) on clinical outcomes and healthcare resource utilization (HCRU) among women with uUTI. Eligible patients were female, aged ≥12 years, with an outpatient diagnosis of uUTI and one or more empirically prescribed oral antibiotics within ±5 days of diagnosis (index). Adjusted risk ratios (aRR) of adverse clinical outcomes were assessed ≤12 months post index (observation period), and rate ratios of all-cause and UTI-related HCRU during the index episode and observation period were observed. Of 376,004 patients, 62,873 (16.7%) experienced TF, while 313,131 (83.3%) did not. Patients with TF had significantly higher risk (aRR [95% CI]) of recurrent UTI (1.23 [1.21, 1.25]) and acute pyelonephritis (1.77 [1.70, 1.84]) (both *p* < 0.001) versus those without. Patients with TF had significantly greater all-cause and UTI-related HCRU versus patients without. Overall, patients with TF had significantly higher risk of adverse outcomes and greater HCRU than patients without TF.

## 1. Introduction

Uncomplicated urinary tract infections (uUTIs), predominantly caused by *Escherichia coli*, are the most commonly diagnosed outpatient infections in women in the United States (US), with >50% of women experiencing one or more episodes in their lifetime [1,2]. uUTIs are typically treated empirically with broad-spectrum antibiotics, which can lead to suboptimal outcomes for patients, for example when an inappropriate antibiotic class is prescribed [3,4,5]. The management of uUTIs is becoming increasingly challenging due to the rise in antibiotic resistance [6]. Consequently, patients have an increased risk of recurrent uUTI episodes and/or treatment failure (TF) [7,8]. In the literature, definitions of TF vary but broadly include uUTI recurrence, uUTI-associated hospitalization or an additional antibiotic prescription within a certain time period (typically ≤28 days following treatment) [9]. A retrospective study of patients with empirically treated uUTI reported a TF rate of 34.3% among patients who received an inappropriate antibiotic, compared with 18.9% of patients who received an appropriate antibiotic [10]. Two other retrospective database studies conducted in the US reported TF rates of 12.3% and 15.2% among female patients with a uUTI [11,12,13].

Antibiotic TF is associated with greater healthcare resource utilization (HCRU, such as urinary tract infection [UTI]-related emergency department [ED] visits, ambulatory care claims and pharmacy claims) and treatment-related costs (including total and uUTI-related healthcare costs), compared with uUTI episodes without TF [11,14]. While short-term clinical outcomes in patients with uUTI-related TF have been evaluated previously, there is a lack of data describing the long-term outcomes for patients with TF. Furthermore, the economic burden on female patients and the US healthcare system associated with uUTI-related TF must be further explored to elucidate unmet needs in this population. We previously conducted a retrospective, observational study, reporting a TF incidence rate of 167.2 per 1000 (16.7%) female patients with uUTI empirically prescribed antibiotics [15]. Antibiotic use in the previous 12 months, UTI recurrence, and location of care were identified as key patient-level risk factors. To better understand the burden of TF in uUTI, we conducted a secondary analysis evaluating the risk of adverse clinical outcomes, as well as HCRU rates and costs associated with TF within 28 days of treatment and over the 12-month observation period.

## 2. Materials and Methods

### 2.1. Study Design

**Figure 1:**
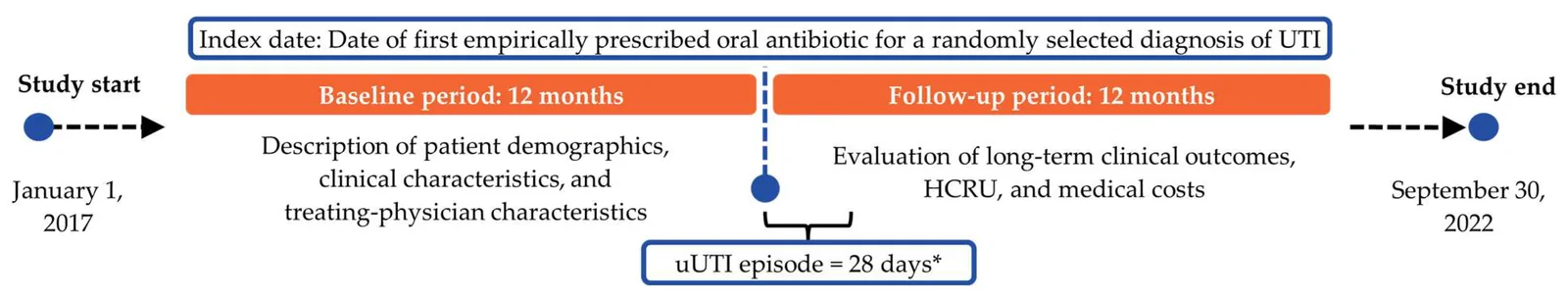
Study design. * For patients with TF during the index uUTI episode, the episode was extended to 28 days after the date of the first observed TF. HCRU, healthcare resource utilization; TF, treatment failure; UTI, urinary tract infection; uUTI, uncomplicated urinary tract infection.

Details of the study design have been published previously [15]. Briefly, this retrospective, observational cohort study used data from Optum’s de-identified Electronic Health Record dataset (Optum EHR; January 2017–September 2022). The study design is shown in Figure 1. The index date was the earliest empiric oral antibiotic prescription occurring within ±5 days of a randomly selected UTI diagnosis (the index diagnosis). The 12 months prior to the index date defined the baseline period and the 28 days following the index date (including the index date) defined the index episode. For patients with TF, the index episode was extended to 28 days after the date of the first observed TF.

### 2.2. Study Population

Eligible patients were female, aged ≥12 years, with ≥1 outpatient or ED uUTI diagnosis and ≥1 oral antibiotic prescription. Key exclusion criteria included antibiotic susceptibility test results <14 days prior to or on the index date, evidence of a complicated UTI (cUTI) [16] during the baseline period or on the index date, hospitalization <28 days prior to or on the index date, or nursing home residence during the baseline period. Full inclusion and exclusion criteria are listed in Table S1.

Patients were grouped into two cohorts: patients who experienced TF (TF cohort) and those who did not experience TF (no-TF cohort). TF was defined as a new or repeat oral antibiotic prescription; administration of an IV antibiotic; or acute UTI diagnosis <28 days following the index date. Acute UTI diagnosis was defined as a primary diagnosis of UTI in an ED or inpatient setting (excluding the index uUTI diagnosis).

### 2.3. Study Outcomes

Patient demographics and clinical characteristics were assessed during the baseline period. Adverse clinical outcomes—recurrent UTI and uUTI complications—were evaluated over the observation period (12 months post index [including the index date]). Recurrent UTI was defined as ≥3 UTI episodes within 12 months (including the index episode), ≥2 UTI episodes within 6 months (including the index episode), or a diagnosis of recurrent UTI as captured by natural language processing (NLP) in the Optum EHR dataset. uUTI complications were captured using International Classification of Diseases, Tenth Edition, Clinical Modification (ICD-10-CM) diagnosis codes and by NLP in the Optum EHR dataset. Complications, assessed separately and as a composite outcome, included acute pyelonephritis (AP; ICD-10-CM: N10) and bacteremia/Gram-negative sepsis (ICD-10-CM R78.81, A41.5).

All-cause and UTI-related HCRU and medical costs were evaluated for the index episode and during the observation period. HCRU included all-cause and UTI-related hospitalizations (including length of stay), ED visits, and outpatient visits (including urgent care, office or clinic visits, ambulatory services utilization, and telephone or online consultation).

Mean monthly medical costs per patient (including hospitalization, ED visit, and outpatient visit costs) were imputed based on unit cost estimates among patients in the US. Unit cost estimates (USD) for an all-cause and UTI-related hospitalization were USD 22,543 in 2017 [17] and USD 6425 in 2011 [18], respectively. Estimates for an ED visit were USD 3516 in 2016 [19] and USD 2598 in 2008 [20], and for an outpatient visit they were USD 478 in 2017 [17] and USD 300 in 2013 [21]. All costs were inflation-adjusted to 2022 USD based on the medical care component of the Consumer Price Index. HCRU and costs were identified as UTI-related if the visit or encounter was associated with a diagnosis code for UTI (ICD-10-CM: N30.0x, N30.9x, and N39.0).

### 2.4. Statistical Analysis

The number of patients with TF was reported as a proportion of the total population. Risks of recurrent UTI, AP, and bacteremia/Gram-negative sepsis were compared between patients with and without TF using univariable and multivariable regression models with Poisson distribution to estimate risk ratios (RR), 95% CIs and *p*-values, using robust standard errors. Multivariable models included adjustment for demographics and clinical characteristics with a standardized difference >10% between the two cohorts.

Incidence rates of each HCRU type were reported per 100 patient-months for the index uUTI episode and the 12-month follow-up period. HCRU was compared between cohorts using univariable and multivariable regression models with negative binomial distribution; 95% CIs, and *p*-values were generated using non-parametric bootstrap procedures with 499 replications. Medical costs were compared between patients with and without TF using generalized linear models with a gamma distribution and log-link function to account for the right-skewed distribution of costs and expected substantial proportion of zero values. For cost outcomes with >5% zero values, a logistic regression was used to estimate the marginal probability of non-zero costs, and a generalized linear model with a gamma distribution and log-link function was used to estimate the predicted cost ratio conditioned on the probability of non-zero costs. Multivariable models for HCRU and costs adjusted for age, race, region, and clinical characteristics with a standardized difference >20% between the two cohorts.

## 3. Results

### 3.1. Incidence of TF

Patient disposition and baseline characteristics have been published previously [15]. Among 376,004 patients included, 62,873 (16.7%) experienced TF (TF cohort). The remaining 313,131 patients (83.3%) did not experience TF (no-TF cohort).

### 3.2. Long-Term Clinical Outcomes

**Figure 2:**
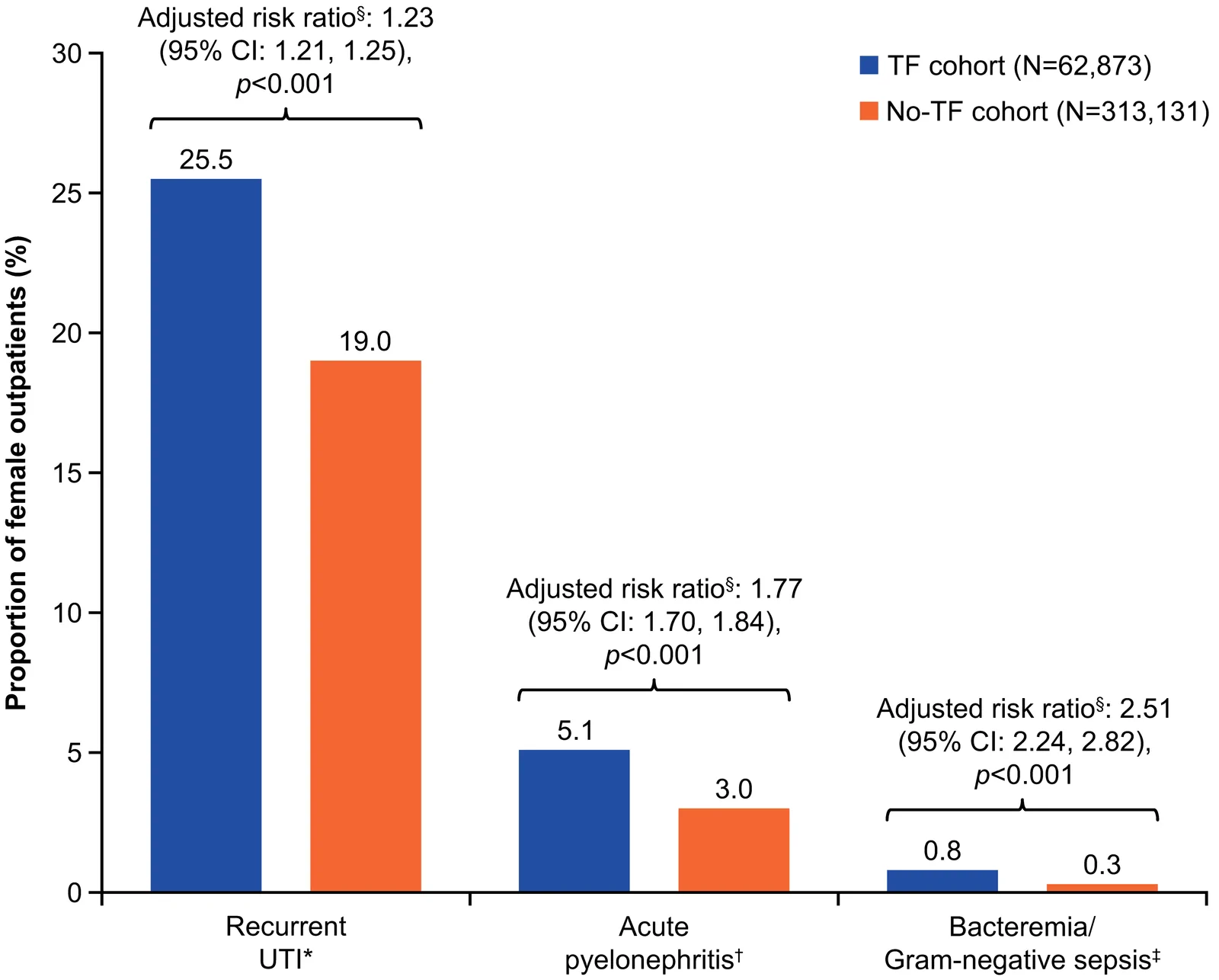
Risk of clinical outcomes during the 12-month observation period among female outpatients with uUTI: TF versus no-TF of empirically prescribed oral antibiotic treatment. * A patient was considered to have recurrent UTI if (1) the patient was diagnosed with recurrent UTI (identified through NLP in the Optum EHR dataset) or (2) the patient had ≥3 UTI episodes during the 12 months post index or ≥2 UTI episodes in the 6 months post index, including the index uUTI diagnosis. ^†^ Acute pyelonephritis was identified using ICD-10-CM diagnosis code N10 or ‘acute pyelonephritis’ as captured by NLP in the Optum EHR dataset. ^‡^ Bacteremia/Gram-negative sepsis was identified using ICD-10-CM diagnosis codes R78.81 (bacteremia) and A41.5 (sepsis due to other Gram-negative organisms, such as *Escherichia coli*) or ‘bacteremia’ or ‘urosepsis’ as captured by NLP in the Optum EHR dataset. ^§^ Adjusted risk ratios were estimated from multivariable generalized linear models with Poisson distribution, adjusting for demographics and the following clinical characteristics with a standardized difference >10% between the TF cohort and no-TF cohort: *Escherichia coli* bacterial pathogen, any antibiotic non-susceptibility for the bacterial pathogen, visit type on the index uUTI diagnosis, pyuria, number of previous UTI episodes, recurrent UTI, nitrite positive urinalysis result, red blood cells present urinalysis result, number of all-cause ED visits, baseline β-lactam use, baseline fluoroquinolone use, baseline use of other antibiotics, number of prescriptions for any oral antibiotic treatment during the baseline period, and baseline history of antibiotic TF. Baseline characteristics collinear with another characteristic already included in the model were not adjusted for (e.g., menopausal status). CI, confidence interval; ED, emergency department; EHR, electronic health record; ICD-10-CM, International Classification of Diseases, 10th Revision, Clinical Modification; NLP, natural language processing; TF, treatment failure; UTI, urinary tract infection; uUTI, uncomplicated urinary tract infection.

During the observation period, patients in the TF cohort had a significantly greater risk of adverse clinical outcomes versus the no-TF cohort. The risks of recurrent UTI and uUTI complications were 23% (adjusted RR [aRR] 1.23; 95% CI 1.21, 1.25; *p* < 0.001) and 80% (aRR 1.80; 95% CI 1.73, 1.87; *p* < 0.001) higher, respectively, in patients with TF versus without TF. Significant between-cohort differences in the risk of individual uUTI complications (all *p* < 0.001) included AP (TF cohort, 5.1%; no-TF cohort, 3.0%; aRR 1.77; 95% CI 1.70, 1.84) and bacteremia/Gram-negative sepsis (TF cohort, 0.8%; no-TF cohort, 0.3%; aRR 2.51; 95% CI 2.24, 2.82; Figure 2).

### 3.3. HCRU and Cost Outcomes

**Figure 3:**
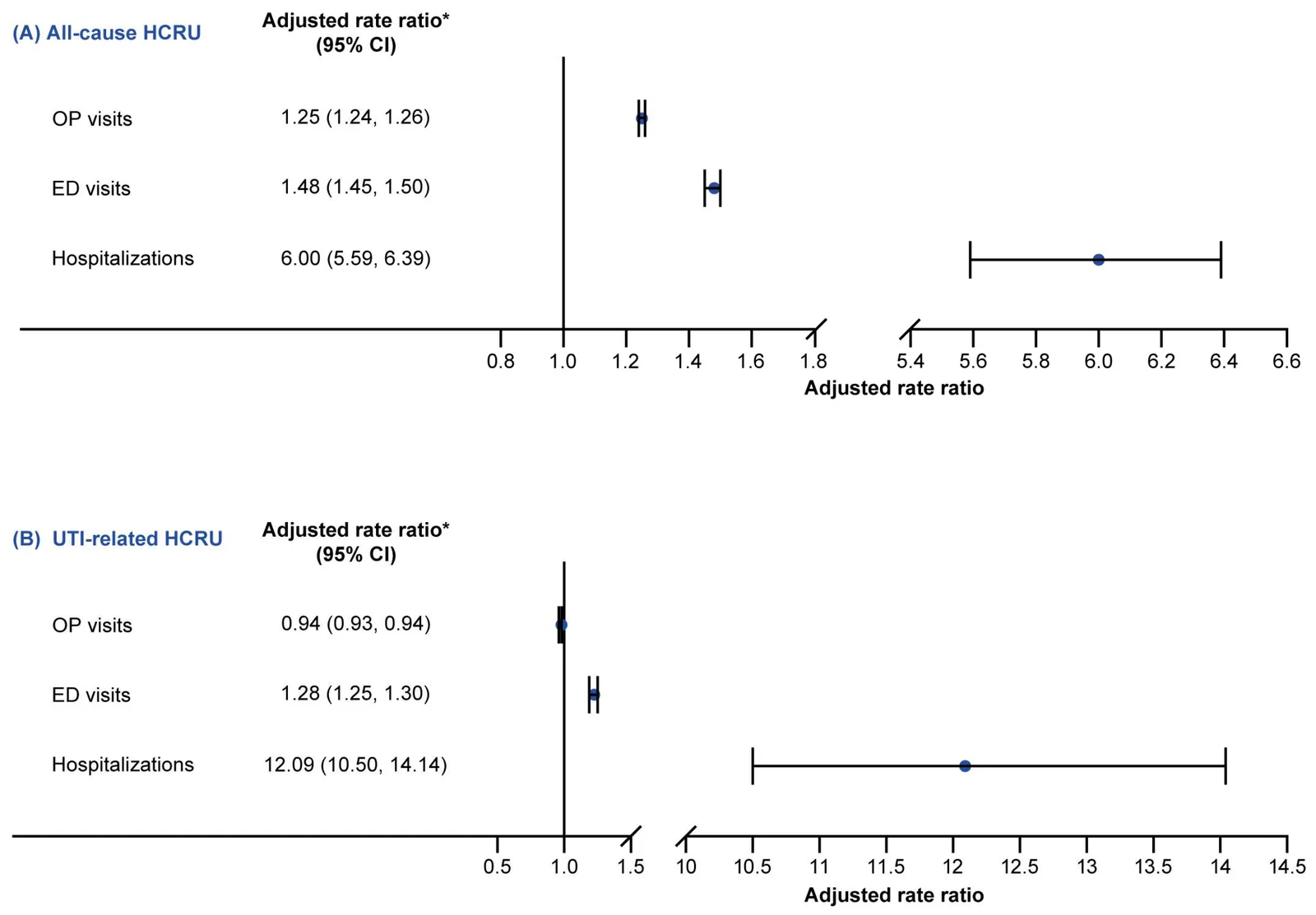
All-cause and UTI-related HCRU during the index uUTI episode among female outpatients with TF versus no-TF of the initial empirically prescribed oral antibiotic treatment. * Adjusted rate ratios were estimated from multivariable generalized linear models with negative binomial distribution to account for overdispersion, adjusting for age, race, and region, as well as the following clinical characteristics with a standardized difference >20% between the TF cohort and no-TF cohort: *Escherichia coli* bacterial pathogen, any antibiotic non-susceptibility for the bacterial pathogen, number of prescriptions for any oral antibiotic treatment in the baseline period, and baseline history of antibiotic TF. Baseline characteristics collinear with another characteristic already included in the model were not adjusted for (e.g., menopausal status). CI, confidence interval; ED, emergency department; HCRU, healthcare resource utilization; OP, outpatient; TF, treatment failure; UTI, urinary tract infection; uUTI, uncomplicated urinary tract infection.

**Figure 4:**
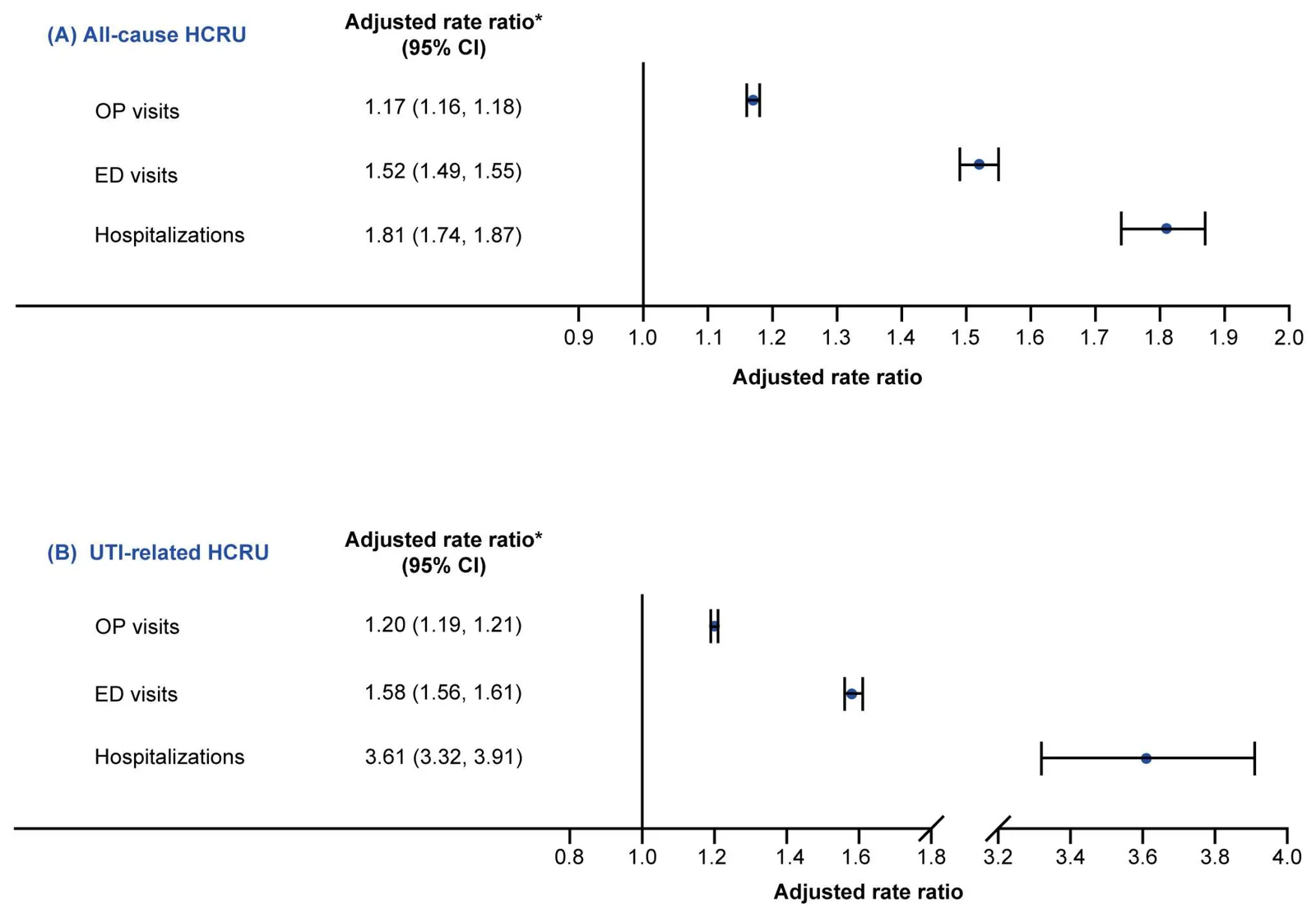
All-cause and UTI-related HCRU during the 12-month observation period among female outpatients with uUTI with TF versus no-TF of the initial empirically prescribed oral antibiotic treatment. * Adjusted rate ratios were estimated from multivariable generalized linear models with negative binomial distribution to account for overdispersion, adjusting for age, race, and region, as well as the following clinical characteristics with a standardized difference >20% between the TF cohort and no-TF cohort: *Escherichia coli* bacterial pathogen, any antibiotic non-susceptibility for the bacterial pathogen, number of prescriptions for any oral antibiotic treatment in the baseline period, and baseline history of antibiotic treatment failure. Baseline characteristics collinear with another characteristic already included in the model were not adjusted for (e.g., menopausal status). CI, confidence interval; ED, emergency department; HCRU, healthcare resource utilization; OP, outpatient; TF, treatment failure; UTI, urinary tract infection; uUTI, uncomplicated urinary tract infection.

During the index uUTI episode, hospitalizations were rare across cohorts; however, the incidence of all-cause hospitalizations was six-fold higher among the TF cohort versus the no-TF cohort (3.68 versus 0.58 per 100 person-months; adjusted incident rate [aIR] 6.00; 95% CI 5.59, 6.39; *p* < 0.001) and the incidence of UTI-related hospitalizations was 12-fold higher (1.25 versus 0.09 per 100 person-months; aIR 12.09; 95% CI 10.50, 14.14; *p* < 0.001; Figure 3). The incidence of ED visits was also significantly higher for those with TF versus those without (all-cause: 30.75 versus 21.34 per 100 person-months; aIR 1.48; 95% CI 1.45, 1.50; UTI-related: 21.36 versus 17.21 per 100 person-months; aIR 1.28; 95% CI 1.25, 1.30; both *p* < 0.001). The rate of all-cause outpatient visits was significantly higher for patients with TF versus without (241.12 versus 187.92 per 100 person-months; aIR 1.25; 95% CI 1.24, 1.26; *p* < 0.001); however, incidence of UTI-related outpatient visits was lower for patients in the TF cohort versus no-TF cohort (86.28 versus 90.87 per 100 person-months; aIR 0.94; 95% CI 0.93, 0.94; *p* < 0.001). All-cause and UTI-related HCRU rates were consistently higher in the TF cohort versus no-TF cohort in the observation period (Figure 4).

Mean all-cause and UTI-related total medical costs per-person per-month during the index uUTI episode were significantly higher for patients in the TF cohort versus no-TF cohort (mean [SD], USD 3440 [USD 5401] versus USD 2108 [USD 3167]; adjusted cost ratio [aCR] 1.66; 95% CI 1.64, 1.68; and USD 1316 [USD 1909] versus USD 1071 [USD 1457]; aCR 1.33; 95% CI 1.31, 1.35; both *p* < 0.001). All-cause and UTI-related costs were consistently higher in the TF cohort versus no-TF cohort for hospitalizations, ED visits, and outpatient visit costs during the index episode and the observation period. Cost data during the index uUTI episode and during the follow-up period are presented in Table S2 and Table S3, respectively.

## 4. Discussion

### 4.1. Key Findings

Antibiotic TF in uUTI is associated with increased clinical and economic burden [5,22]; however, long-term burden (up to 12 months following treatment) is poorly characterized in the literature. This study evaluated the association of antibiotic TF with clinical outcomes, HCRU and costs during the index uUTI episode and the 12 months following treatment. Patients who experienced TF had a significantly higher risk of adverse clinical outcomes during the observation period, including recurrent UTI and uUTI complications, and more than double the risk of experiencing bacteremia/Gram-negative sepsis compared with the no-TF cohort. All-cause and UTI-related HCRU incidence rates were consistently higher among patients with TF versus without, both in the short-term (during the index uUTI episode) and long-term (during the 12-month observation period). In addition, a significantly greater short- and long-term economic burden was observed for patients in the TF cohort versus the no-TF cohort, with the greatest difference seen in UTI-related hospitalization costs, which were 13-fold higher during the index uUTI episode among patients with TF versus without.

### 4.2. Clinical Significance of Results

Antibiotic TF associated with uUTI is closely related to antimicrobial resistance (AMR), a growing global public health concern [6,23], and there is established research on the prevalence of AMR in the US (0.4–35.9% depending on bacterium strain and antimicrobial class) [24,25]. While recurrent UTIs are associated with resistant uropathogens [26], data on long-term clinical outcomes and related complications (such as bacteremia/Gram-negative sepsis) due to TF are limited. To our knowledge, this is the first study to evaluate these outcomes in the context of TF for uUTI.

In the current study, the incidence of recurrent UTI was significantly higher among patients with TF, yet the incidence was also relatively high among those without TF (~20%). Previous studies reported that 19–44% of female patients will experience recurrence within 6 months to a year [27,28]. The incidence of UTI complications in the current study was 5.7% in the TF cohort and 3.2% in the no-TF cohort. Another observational study in the US reported a subsequent diagnosis of urosepsis or pyelonephritis in 1.7% of female patients within 60 days of a uUTI diagnosis [29]. The higher incidence of UTI complications reported in the current study are likely attributable to the extended observation period of 12 months. Together, these findings highlight an urgent need to improve outcomes for patients who experience TF and/or adverse clinical outcomes and the importance of long-term follow-up for patients with uUTI.

The HCRU rates and costs reported during the observation period in the current study were higher for patients with TF versus without. A previous observational study by Ellis et al. (2025) reported significantly higher rates of UTI-related outpatient hospital visits and physician office visits during days 181–365 following a uUTI episode among patients with TF versus those without TF (14.1% versus 9.8% and 16.6% versus 11.9%, respectively; both *p* < 0.001) [13]. In the same time period, total UTI-related mean medical costs were USD 162 more per patient in the TF versus no-TF cohorts (*p* < 0.001) [13]. Similarly, Franklin et al. (2023) reported consistently higher UTI-related total medical, outpatient, and inpatient costs among patients with uUTI TF versus those without during a one-year follow-up period [11]. The current study adds to the limited existing evidence base, providing contemporary data on long-term HCRU and costs associated with antibiotic TF, and further highlights the disproportionate clinical and economic burden on patients who experience TF.

Similar trends of the impact of TF are observed in other bacterial infections. For example, in a post hoc analysis of a randomized, non-inferiority trial of linezolid versus vancomycin in patients with nosocomial pneumonia caused by methicillin-resistant *Staphylococcus aureus*, TF was a significant predictor of greater medical costs compared with those with treatment success (*p* < 0.001) [30]. Furthermore, TF has been reported in patients with acute otitis media (2.2% [95% CI 2.1, 2.2]) [31] and acute sinusitis (3.2% in patients treated with amoxicillin-clavulanate and 2.9% with amoxicillin) [32]. However, there is little evidence describing the burden of TF in these populations. Given the significantly higher clinical and financial burden on patients with versus without uUTI-related TF observed in the current study, further investigations of TF-related outcomes in other common outpatient infections are required to provide a comprehensive understanding of the burden of TF in general.

A wide range of uUTI-associated TF incidence rates have been reported across previous retrospective studies (4.6–23.4%) [12,33,34], likely attributable to differences in TF definitions and the study populations. A recent systematic literature review evaluated uUTI-associated TF definition heterogeneity across 10 observational studies using administrative databases and 11 observational studies using patient-level health record data [9]. Definitions of UTI and TF differed substantially across studies. Among database studies, UTI identification relied primarily on ICD-9 or ICD-10-CM codes, although two studies used International Classification of Primary Care codes and one study required culture-positive UTI confirmation. All 10 database studies included a second antibiotic prescription in their TF definition, but many also incorporated composite endpoints such as IV administration and/or hospitalizations/ED visits. In contrast, studies using health record data employed more clinically nuanced criteria to define TF, including progression to severe or cUTI requiring hospitalization/ED visit, evidence of pyelonephritis, persistent symptoms, or use of a validated symptom assessment questionnaire (the Urinary Tract Infection Symptom Assessment) to determine treatment response. The substantial variation in TF definitions underscores the need for greater standardization to better characterize its impact on patients and healthcare systems.

Overall, the results reported here suggest that access to new oral antimicrobial treatments is needed to improve clinical outcomes and reduce the clinical and economic burden on patients with uUTI. Most recently, gepotidacin, a bactericidal, first-in-class triazaacenaphthylene antibacterial, was approved in March 2025 by the US Food and Drug Administration for the treatment of female patients with uUTI [35]. However, further research is needed to assess the impact of this new class of therapy on long-term patient outcomes and HCRU.

### 4.3. Limitations

The limitations of this study included the lack of culture-proven UTI diagnosis. This may have led to overestimation of uUTI-associated TF if patients received a new prescription for an infection from a different microorganism or if uUTIs were over-diagnosed where patients presented with UTI symptoms in the absence of bacteriuria. However, guidelines do not consistently recommend urine cultures from patients in general practice to diagnose uUTI [36,37], with US primary care studies indicating that cultures are obtained in only 30–57% of patients [38,39,40,41]. Therefore, requiring a culture-proven diagnosis for study entry would have considerably underestimated the real-world occurrence of TF. Furthermore, the definition of TF used in the current study may not directly translate to microbiologically confirmed TF or AMR; therefore, these results should be interpreted cautiously. Additionally, uUTI and cUTI definitions are evolving. The American Urological Association (AUA) and the Infectious Diseases Society of America (IDSA) recently amended these definitions to reclassify male patients with afebrile UTI and no signs or symptoms of systemic infection as uUTI [36,37].

Another limitation is the lack of a standardized TF definition, which may have driven the lower TF incidence rates reported in previous studies. For example, Koh et al. (2023) reported a TF rate of 4.6% in patients with uUTI; however, they had a young population (ages 18–50 years) and strict antibiotic re-prescription criterion, requiring a follow-up visit, the presence of UTI symptoms and a prescription on the same date [34]. In comparison, Franklin et al. (2023) reported a TF rate of 12.3% in adult female patients with an outpatient uUTI diagnosis; TF was defined as ≥1 additional oral antibiotic prescription, IV administration or a new UTI diagnosis in ED or inpatient setting ≤28 days of the antibiotic claim date [11], consistent with the TF definition used in the current study. This limitation could be mitigated by the introduction of a standardized TF definition across observational and clinical trials.

Lastly, UTI-related HCRU and costs included all visits associated with a uUTI diagnosis code, which did not include diagnosis codes for AP or related conditions or diagnosis codes for Gram-negative sepsis with secondary UTI, diagnoses which may have indicated a progression to a more severe UTI or cUTI per the IDSA guidelines (2025) [37]. Therefore, UTI-related HCRU and costs may have been underestimated in this study. Furthermore, exact cost data were not available, and costs were instead estimated based on values reported in the literature. This approach was applied to both cohorts and was not expected to have a differential impact; therefore, the cost ratio still captures the greater financial burden observed among patients with TF versus without TF.

## 5. Conclusions

This study found that female patients with uUTI who experienced TF to empirically prescribed oral antibiotics had significantly greater risk of adverse clinical outcomes, as well as higher rates of all-cause and UTI-related HCRU and costs in the short- and long-term versus patients without TF. Further research to elucidate the factors contributing to TF and identify effective antibiotic prescribing practices is urgently needed to improve clinical outcomes for patients with uUTI and reduce the economic burden associated with TF.

## Data Availability

The datasets during and/or analyzed during the current study are available from the corresponding author on reasonable request.

## Supplementary Materials

The following supporting information can be downloaded at https://www.mdpi.com/article/10.3390/microorganisms14091993/s1. Table S1. Key eligibility criteria; Table S2. Monthly medical costs during the index uUTI episode among female outpatients with uUTI with TF versus no-TF to the initial empirically prescribed oral antibiotic treatment; Table S3. Monthly medical costs during the 12-month observation period among female outpatients with uUTI with TF versus no-TF to the initial empirically prescribed oral antibiotic treatment.

## References

[1] Medina M., Castillo-Pino E. (2019). An introduction to the epidemiology and burden of urinary tract infections. Ther. Adv. Urol..

[2] Vautrin N., Alexandre K., Pestel-Caron M., Bernard E., Fabre R., Leoz M., Dahyot S., Caron F.J.M.S. (2023). Contribution of Antibiotic Susceptibility Testing and CH Typing Compared to Next-Generation Sequencing for the Diagnosis of Recurrent Urinary Tract Infections Due to Genetically Identical *Escherichia coli* Isolates: A Prospective Cohort Study of Cystitis in Women. Microbiol. Spectr..

[3] Kaye K.S., Belley A., Barth P., Lahlou O., Knechtle P., Motta P., Velicitat P. (2022). Effect of Cefepime/Enmetazobactam vs Piperacillin/Tazobactam on Clinical Cure and Microbiological Eradication in Patients With Complicated Urinary Tract Infection or Acute Pyelonephritis: A Randomized Clinical Trial. JAMA.

[4] Seo Y.B., Lee J., Kim Y.K., Lee S.S., Lee J.A., Kim H.Y., Uh Y., Kim H.S., Song W. (2017). Randomized controlled trial of piperacillin-tazobactam, cefepime and ertapenem for the treatment of urinary tract infection caused by extended-spectrum beta-lactamase-producing *Escherichia coli*. BMC Infect. Dis..

[5] Butler A.M., Durkin M.J., Keller M.R., Ma Y., Dharnidharka V.R., Powderly W.G., Olsen M.A. (2021). Risk of antibiotic treatment failure in premenopausal women with uncomplicated urinary tract infection. Pharmacoepidemiol. Drug Saf..

[6] Friedman N.D., Temkin E., Carmeli Y. (2016). The negative impact of antibiotic resistance. Clin. Microbiol. Infect..

[7] Raphael E., Argante L., Cinconze E., Nannizzi S., Belmont C., Mastrangelo C.F., Allegretti Y.H., Pellegrini M., Schmidt J.E. (2024). Incidence and Recurrence of Urinary Tract Infections Caused by Uropathogenic *Escherichia coli*: A Retrospective Cohort Study. Res. Rep. Urol..

[8] Naber K.G., Wagenlehner F., Kresken M., Cheng W.Y., Catillon M., Duh M.S., Yu L., Khanal A., Mulgirigama A., Joshi A.V. (2023). *Escherichia coli* resistance, treatment patterns and clinical outcomes among females with uUTI in Germany: A retrospective physician-based chart review study. Sci. Rep..

[9] Luck M.E., Martin A., Punja S., Kamar J., Zuchinali P., Edgecomb A.G., Ellis J.J. (2026). Definitions and rates of treatment failure in females with uncomplicated urinary tract infection: A systematic literature review. J. Antimicrob. Chemother..

[10] Dunne M.W., Puttagunta S., Aronin S.I., Brossette S., Murray J., Gupta V. (2022). Impact of empirical antibiotic therapy on outcomes of outpatient urinary tract infection due to nonsusceptible *Enterobacterales*. Microbiol. Spectr..

[11] Franklin M., Emden M.R., Kautz S., Nguyen A.T.H., Sacks N.C., Ju S., Mitrani-Gold F.S., Joshi A.V., Preib M.T. (2023). 2839. Cost Burden of Patients with Oral Antibiotic Treatment Failure for Uncomplicated Urinary Tract Infection in the United States. Open Forum Infect. Dis..

[12] Franklin M., Sacks N.C., Emden M., Kautz S., Joshi A.V., Mitrani-Gold F.S., Preib M. (2023). EE548 Healthcare Resource Use and Costs Associated with Oral Antibiotic Treatment Failure in Uncomplicated Urinary Tract Infection in the US. Value Health.

[13] Ellis J.J., Iyengar A., Bandi H., Niesen M.J.M., Calay E.S., Wagner T.E., Preib M.T., Edgecomb A.G., Luck M.E. (2025). Impact of empirical treatment failure on health care resource utilization and costs among female patients with uncomplicated urinary tract infections in a US-based Integrated Health Delivery Network. J. Manag. Care Spec. Pharm..

[14] Moon R.C., Marijam A., Mitrani-Gold F.S., Gibbons D.C., Kartashov A., Rosenthal N.A., Joshi A.V. (2022). Treatment patterns, healthcare resource use, and costs associated with uncomplicated urinary tract infection among female patients in the United States. PLoS ONE.

[15] Fromer D.L., Luck M.E., Cheng W.Y., Mahendran M., da Costa W.L., Pinaire M., Duh M.S., Preib M.T., Ellis J.J. (2025). Risk Factors for Empiric Treatment Failure in US Female Outpatients with Uncomplicated Urinary Tract Infection: An Observational Study. J. Gen. Intern. Med..

[16] Sabih A., Leslie S.W. (2023). Complicated Urinary Tract Infections. StatPearls [Internet].

[17] Moses M.W., Pedroza P., Baral R., Bloom S., Brown J., Chapin A., Compton K., Eldrenkamp E., Fullman N., Mumford J.E. (2019). Funding and services needed to achieve universal health coverage: Applications of global, regional, and national estimates of utilisation of outpatient visits and inpatient admissions from 1990 to 2016, and unit costs from 1995 to 2016. Lancet Public Health.

[18] Simmering J.E., Tang F., Cavanaugh J.E., Polgreen L.A., Polgreen P.M. (2017). The Increase in Hospitalizations for Urinary Tract Infections and the Associated Costs in the United States, 1998–2011. Open Forum Infect. Dis..

[19] Lane B.H., Mallow P.J., Hooker M.B., Hooker E. (2020). Trends in United States emergency department visits and associated charges from 2010 to 2016. Am. J. Emerg. Med..

[20] Caldwell N., Srebotnjak T., Wang T., Hsia R. (2013). “How much will I get charged for this?” Patient charges for top ten diagnoses in the emergency department. PLoS ONE.

[21] Sammon J., Ghani K., Sukumar S., Abdo A., Djahangirian O., Jeong W., Staley S., Peabody J., Shariate S., Karakiewicz P. (2013). 1062 Socioeconomic Trends and Utilization in the Emergency Department Treatment of Urinary Tract Infections. J. Urol..

[22] Dunne M.W., Aronin S.I., Das A.F., Akinapelli K., Zelasky M.T., Puttagunta S., Boucher H.W. (2023). Sulopenem or Ciprofloxacin for the Treatment of Uncomplicated Urinary Tract Infections in Women: A Phase 3, Randomized Trial. Clin. Infect. Dis..

[23] Anthony W.E., Burnham C.D., Dantas G., Kwon J.H. (2021). The Gut Microbiome as a Reservoir for Antimicrobial Resistance. J. Infect. Dis..

[24] Monari C., Onorato L., Cornelli A., Macera M., Allegorico E., Ferraro A., Nasta C., Florio M.T., Russo K., Bianco P. (2025). Prevalence and outcomes of Urinary tract infections caused by Enterobacterales resistant to third-generation cephalosporins in the Emergency Department: Results from UTILY cohort, a prospective multicentre study. Infection.

[25] Bruins M.J., Eijkelkamp-Biesterbos L., Meutstege A.M., Dos Santos C.O. (2025). *Escherichia coli* antimicrobial resistance in acute urinary tract infection lower than reported in Dutch national surveillance database. PLoS ONE.

[26] Fromer D.L., Cheng W.Y., Gao C., Mahendran M., Hilts A., Duh M.S., Joshi A.V., Mulgirigama A., Mitrani-Gold F.S. (2024). Likelihood of Antimicrobial Resistance in Urinary *E. coli* Isolates Among US Female Patients with Recurrent Versus Non-Recurrent uUTI. Urology.

[27] Brubaker L., Carberry C., Nardos R., Carter-Brooks C., Lowder J.L. (2018). American Urogynecologic Society Best-Practice Statement: Recurrent Urinary Tract Infection in Adult Women. Female Pelvic Med. Reconstr. Surg..

[28] Aggarwal N., Leslie S.W. (2026). Recurrent Urinary Tract Infections. StatPearls [Internet].

[29] Bradley M.S., Ford C., Stagner M., Handa V., Lowder J. (2022). Incidence of urosepsis or pyelonephritis after uncomplicated urinary tract infection in older women. Int. Urogynecology J..

[30] Niederman M.S., Chastre J., Solem C.T., Wan Y., Gao X., Myers D.E., Haider S., Li J.Z., Stephens J.M. (2014). Health economic evaluation of patients treated for nosocomial pneumonia caused by methicillin-resistant *Staphylococcus aureus*: Secondary analysis of a multicenter randomized clinical trial of vancomycin and linezolid. Clin. Ther..

[31] Frost H.M., Bizune D., Gerber J.S., Hersh A.L., Hicks L.A., Tsay S.V. (2022). Amoxicillin Versus Other Antibiotic Agents for the Treatment of Acute Otitis Media in Children. J. Pediatr..

[32] Savage T.J., Kronman M.P., Sreedhara S.K., Lee S.B., Oduol T., Huybrechts K.F. (2023). Treatment Failure and Adverse Events After Amoxicillin-Clavulanate vs Amoxicillin for Pediatric Acute Sinusitis. JAMA.

[33] Mitzner T.M., Eid K.M., Hughson D.M., Jameson A.P., Dumkow L.E. (2025). Cefdinir Versus Cephalexin for the Treatment of Uncomplicated Urinary Tract Infections. Open Forum Infect. Dis..

[34] Koh S.W.C., Ng T.S.M., Loh V.W.K., Goh J.C., Low S.H., Tan W.Z., Wong H.C., Durai P., Sun L.J., Young D. (2023). Antibiotic treatment failure of uncomplicated urinary tract infections in primary care. Antimicrob. Resist. Infect. Control.

[35] GSK BluJepa (Gepotidacin) [Package Insert]. http://www.accessdata.fda.gov/drugsatfda_docs/label/2025/218230s000lbl.pdf.

[36] Ackerman A.L., Bradley M.S., D’Anci K.E., Hickling D., Kim S.K., Kirkby E. Updates to Recurrent Uncomplicated Urinary Tract Infections in Women: AUA/CUA/SUFU Guideline (2025). https://www.auanet.org/guidelines-and-quality/guidelines/recurrent-uti.

[37] IDSA IDSA 2025 Guideline Update on Complicated Urinary Tract Infections. https://www.idsociety.org/practice-guideline/complicated-urinary-tract-infections/.

[38] Carey T.S., Weis K. (1990). Diagnostic testing and return visits for acute problems in prepaid, case-managed Medicaid plans compared with fee-for-service. Arch. Intern. Med..

[39] Johnson J.D., O’Mara H.M., Durtschi H.F., Kopjar B. (2011). Do urine cultures for urinary tract infections decrease follow-up visits?. J. Am. Board Fam. Med..

[40] Wigton R.S., Longenecker J.C., Bryan T.J., Parenti C., Flach S.D., Tape T.G. (1999). Variation by specialty in the treatment of urinary tract infection in women. J. Gen. Intern. Med..

[41] Bruxvoort K.J., Bider-Canfield Z., Casey J.A., Qian L., Pressman A., Liang A.S., Robinson S., Jacobsen S.J., Tartof S.Y. (2020). Outpatient Urinary Tract Infections in an Era of Virtual Healthcare: Trends From 2008 to 2017. Clin. Infect. Dis..

