# Supplementary material for: When Empiric Therapy Fails: Clinical and Economic Consequences in uUTIs Among US Women
Source: Microorganisms. 2026 Sep 9;14(9):1993. doi: 10.3390/microorganisms14091993 (PMC13609793; doi:10.3390/microorganisms14091993)
Supplement: Supplementary file 1 [file microorganisms-14-01993-s001.zip › microorganisms-4527363-supplementary.pdf]

Title: When Empiric Therapy Fails: Clinical and Economic Consequences in uUTIs Among US Women

Authors: Debra L. Fromer [1] , Jeffrey J. Ellis [2] , Rose Chang [3], Meghan E. Luck [2], Megan Pinaire [3], Malena Mahendran [3], Madison Preib [2], Mei Sheng Duh [3] and Amy Edgecomb [2,*]

Affiliations:

1 Hackensack University Medical Center, Hackensack, NJ 07601, USA
2 GSK, Collegeville, PA 19426, USA
3 Analysis Group, Inc., Boston, MA 02199, USA
* Correspondence: amy.x.guisinger@gsk.com

Supplementary tables/figures: 3 tables

Keywords: Clinical outcomes; Economic burden; Healthcare resource utilization; Antibiotic treatment failure; Uncomplicated urinary tract infection.

Table S1. Key eligibility criteria.

Key inclusion criteria	Key exclusion criteria
• Female<br>• ≥12 years of age on the index date*<br>• Documented diagnosis code for UTI (ICD-10-CM codes: N30.00, N30.01, N30.90, N30.91, or N39.0) in an outpatient ambulatory or ED setting between January 1, 2018 and September 30, 2020<br>• Oral antibiotic prescription within ±5 days of diagnosis<br>• Oral treatment with nitrofurantoin, trimethoprim-sulfamethoxazole (separately or in combination), fluoroquinolones, fosfomycin, and/or beta-lactams<br>• ≥12 months of EHR activity[†] prior to the index date, defined as the baseline period<br>• ≥12 months of EHR activity[†] after the index date	• Antibiotic susceptibility test results of the index UTI isolate within 14 days prior to or on the index date<br>• Evidence of complicated UTI[1] during the baseline period or on the index date<br>○ Pregnancy, identified using (1) ICD-10-CM diagnosis codes, (2) ICD-10-PCS codes, (3) CPT codes, and (4) identification of “pregnancy” and related terms as captured by NLP in the Optum EHR dataset<br>○ Urological abnormalities<br>○ Uncontrolled or complicated diabetes, identified using ICD-10-CM diagnosis codes and HbA1c ≥8%<br>○ Severe renal dysfunction<br>○ Immunosuppressed or treated with immunosuppressive therapy<br>○ Urological or nephrological procedures (e.g., catheter, surgery) within 28 days prior to or on the index date and/or ureteral stent procedure during the baseline period<br>○ Intravenous antibiotics within 28 days prior to or on the index date<br>• Hospitalization within 28 days prior to or on the index date<br>• Resident of a nursing home or long-term care facility during the baseline period[‡] or on the index date

[1]Sabih A, Leslie SW. Complicated Urinary Tract Infections. In: StatPearls [Internet]. Treasure Island (FL): StatPearls Publishing; 2023.

*The index date was the date of initial oral antibiotic prescription for the index UTI diagnosis.
[†]EHR activity was identified via the First Month Active and Last Month Active fields of the patient data set, which includes patient demographic details (e.g., birth year, gender, race). The First Month Active field is defined by Optum as the earliest month and year with a recorded healthcare activity event in the dataset. Patients had an active month in the First Month Active field that is ≥12 months prior to the index date. The Last Month Active field is defined as the latest month and year with a recorded healthcare activity event. Patients had an active month in the Last Month Active field that is ≥12 months following their index date.
[‡]12 months prior to the index date.

CPT, current procedural terminology; ED, emergency department; EHR, electronic health record; HbA1c, hemoglobin A1c; ICD-10-CM, International Classification of Diseases, Tenth Edition, Clinical Modification; ICD-10-PCS, International Classification of Diseases, Tenth Edition, Procedure Coding System; NLP, natural language processing; UTI, urinary tract infection.

Table S2. Monthly medical costs during the index uUTI episode among female outpatients with uUTI with TF versus no-TF to the initial empirically prescribed oral antibiotic treatment.

	TF cohort [A] N=62,873	No-TF cohort [B] N=313,131	Unadjusted cost ratio† (95% CI)‡ [A]/[B]	*p*-value‡	Adjusted cost ratio§ (95% CI)‡ [A]/[B]	*p*-value‡
**Observation period,* months, mean ± SD (median)**	1.17 ± 0.24 (1)	0.92 ± 0.00 (1)	–	–	–	–
**Medical costs, PPPM, $US 2022**						
**All-cause medical costs, mean ± SD (median)**	3440 ± 5401 (1835)	2108 ± 3167 (1016)	1.65 (1.63, 1.67)	<0.001	1.66 (1.64, 1.68)	<0.001
Hospitalization costs	738 ± 4150 (0)	136 ± 2092 (0)	5.40 (5.05, 5.80)	<0.001	4.72 (4.35, 5.11)	<0.001
ED visit costs	1195 ± 2224 (0)	830 ± 1903 (0)	1.52 (1.50, 1.55)	<0.001	1.44 (1.42, 1.46)	<0.001
OP visit costs¶	1506 ± 1473 (1116)	1142 ± 1156 (1016)	1.30 (1.29, 1.32)	<0.001	1.32 (1.31, 1.33)	<0.001
**UTI-related medical costs,** mean ± SD (median)**	1316 ± 1909 (405)	1071 ± 1457 (419)	1.30 (1.29, 1.32)	<0.001	1.33 (1.31, 1.35)	<0.001
Hospitalization costs	110 ± 977 (0)	8 ± 279 (0)	14.99 (12.91, 17.21)	<0.001	13.78 (11.56, 16.39)	<0.001
ED visit costs	833 ± 1642 (0)	671 ± 1550 (0)	1.34 (1.32, 1.37)	<0.001	1.30 (1.27, 1.32)	<0.001
OP visit costs¶	373 ± 307 (355)	392 ± 247 (419)	0.96 (0.96, 0.97)	<0.001	0.98 (0.97, 0.98)	<0.001

*The index uUTI episode was defined as the 28 days following (and including) the index date. For patients with treatment failure during the index uUTI episode, the episode was extended to 28 days after the date of first observed treatment failure.

†Unadjusted cost ratios were estimated from generalized linear models with gamma distribution and log-link function. For cost outcomes with >5% zero values, a logistic regression was used to estimate the marginal probability of non-zero costs and a generalized linear model with a gamma distribution and log-link function was used to estimate the predicted cost ratio conditioned on the probability of non-zero costs.

‡95% CIs and *p*-values were generated using non-parametric bootstrap procedures with 499 replications.

§Adjusted cost ratios were estimated from multivariable generalized linear models with gamma distribution and log-link function, controlling for age, race, and region, as well as the following clinical characteristics with a standardized difference >20% between the treatment failure cohort and no treatment failure cohort: *Escherichia coli* bacterial pathogen, any antibiotic non-susceptibility of the bacterial pathogen, number of prescriptions for any oral antibiotic treatment in the baseline period, and baseline history of antibiotic treatment failure. Among collinear characteristics, only one characteristic was included for adjustment (e.g., age was included as opposed to menopausal status). For cost outcomes with >5% zero values, a logistic regression was used to estimate the marginal probability of non-zero costs and a generalized linear model with a gamma distribution and log-link function was used to estimate the predicted cost ratio conditioned on the probability of non-zero costs.

¶OP visits included urgent care visits, ambulatory services, office or clinic visits, telephone/online visits.

**UTI-related costs were identified as visits associated with an ICD-10-CM UTI diagnosis code (i.e., N30.0x, N30.9x, and N39.0).

CI, confidence interval; ED, emergency department; ICD-10-CM, International Classification of Diseases, 10th Revision, Clinical Modification; OP, outpatient; PPPM, per patient per month; SD, standard deviation; TF, treatment failure; US, United States; UTI, urinary tract infection; uUTI, uncomplicated urinary tract infection.

Table S3. Monthly medical costs during the 12-month observation period among female outpatients with uUTI with TF versus no-TF to the initial empirically prescribed oral antibiotic treatment.

Medical costs, PPPM, $US 2022	TF cohort [A] N=62,873	No TF cohort [B] N=313,131	Unadjusted cost ratio* (95% CI)† [A]/[B]	*p*-value†	Adjusted cost ratio‡ (95% CI)† [A]/[B]	*p*-value†
**All-cause medical costs, mean ± SD (median)**	1117 ± 1548 (628)	795 ± 1192 (436)	1.41 (1.39, 1.42)	<0.001	1.43 (1.41, 1.45)	<0.001
Hospitalization costs	254 ± 990 (0)	139 ± 741 (0)	1.83 (1.76, 1.89)	<0.001	1.79 (1.72, 1.85)	<0.001
ED visit costs	265 ± 532 (0)	174 ± 400 (0)	1.53 (1.50, 1.56)	<0.001	1.47 (1.45, 1.50)	<0.001
OP visit costs§	598 ± 694 (399)	482 ± 573 (307)	1.24 (1.23, 1.25)	<0.001	1.28 (1.26, 1.29)	<0.001
**UTI-related medical costs,¶ mean ± SD (median)**	152 ± 224 (64)	96 ± 146 (32)	1.58 (1.56, 1.60)	<0.001	1.60 (1.58, 1.62)	<0.001
Hospitalization costs	18 ± 128 (0)	4 ± 63 (0)	4.44 (4.09, 4.79)	<0.001	3.61 (3.24, 3.96)	<0.001
ED visit costs	90 ± 175 (0)	57 ± 134 (0)	1.58 (1.55, 1.61)	<0.001	1.52 (1.50, 1.55)	<0.001
OP visit costs§	44 ± 45 (32)	35 ± 29 (32)	1.26 (1.25, 1.27)	<0.001	1.28 (1.27, 1.29)	<0.001

*Unadjusted cost ratios were estimated from generalized linear models with gamma distribution and log-link function. For cost outcomes with >5% zero values, a logistic regression was used to estimate the marginal probability of non-zero costs and a generalized linear model with a gamma distribution and log-link function was used to estimate the predicted cost ratio conditioned on the probability of non-zero costs.
†95% CIs and *p*-values were generated using non-parametric bootstrap procedures with 499 replications.
‡Adjusted cost ratios were estimated from multivariable generalized linear models with gamma distribution and log-link function, controlling for age, race, and region, as well as the following clinical characteristics with a standardized difference >20% between the treatment failure cohort and no treatment failure cohort: *Escherichia coli* bacterial pathogen, any antibiotic non-susceptibility of the bacterial pathogen, number of prescriptions for any oral antibiotic treatment in the baseline period, and baseline history of antibiotic treatment failure. Among collinear characteristics, only one characteristic was included for adjustment (e.g., age was included as opposed to menopausal status). For cost outcomes with >5% zero values, a logistic regression was used to estimate the marginal probability of non-zero costs and a generalized linear model with a gamma distribution and log-link function was used to estimate the predicted cost ratio conditioned on the probability of non-zero costs.
§OP visits included urgent care visits, ambulatory services, office or clinic visits, telephone/online visits.
¶UTI-related costs were identified as visits associated with an ICD-10-CM UTI diagnosis code (i.e., N30.0x, N30.9x, and N39.0).

CI, confidence interval; ED, emergency department; ICD-10-CM, International Classification of Diseases, 10th Revision, Clinical Modification; OP, outpatient; PPPM, per patient per month; SD, standard deviation; TF; treatment failure; US, United States; UTI, urinary tract infection; uUTI, uncomplicated urinary tract infection.
